# Disease Activity and Tendency to Relapse in ANCA-Associated Vasculitis Are Reflected in Neutrophil and Intermediate Monocyte Frequencies

**DOI:** 10.1155/2024/6648265

**Published:** 2024-01-03

**Authors:** Sofia Smargianaki, Evelina Elmér, Sandra Lilliebladh, Sophie Ohlsson, Åsa Pettersson, Thomas Hellmark, Åsa CM Johansson

**Affiliations:** ^1^Division of Hematology and Transfusion Medicine, Department of Laboratory Medicine, Lund University and Clinical Immunology and Transfusion Medicine, Skåne University Hospital, Lund, Sweden; ^2^Nephrology, Department of Clinical Sciences Lund, Lund University, Skåne University Hospital, Lund, Sweden; ^3^Division of Hematology and Transfusion Medicine, Department of Laboratory Medicine, Lund University and Clinical Genetics and Pathology, Skåne University Hospital, Lund, Sweden

## Abstract

Antineutrophil cytoplasmic antibody (ANCA)-associated vasculitis (AAV) is a group of autoimmune diseases with inflammation affecting small blood vessels and includes granulomatosis with polyangiitis (GPA) and microscopic polyangiitis (MPA). In this study, we investigated granulocyte and monocyte subsets in a large cohort of AAV patients with emphasis on disease activity and tendency to relapse. A cohort of 105 patients with GPA or MPA and 126 healthy controls (HCs) were included. Clinical and laboratory data were collected for all patients, including disease activity, tendency to relapse, and pharmacological treatment. Using flow cytometry, circulating eosinophils, basophils, neutrophils, and monocytes were assessed. The monocytes were subdivided into classical (CD14^++^CD16^−^), intermediate (CD14^++^CD16^+^), and nonclassical (CD14^−^CD16^+^) monocytes. Mature (CD16^high^) or newly released (CD16^dim^) neutrophils were defined, as well as the frequency of CD177^+^ neutrophils. AAV patients displayed increased frequencies of intermediate monocytes, mature and newly released neutrophils, and an expanded population of CD177^+^ neutrophils compared to HC. MPA patients differed from GPA patients in terms of lower frequency of classical monocytes. No differences in cell frequencies regarding ANCA phenotype were observed. Paired data from 23 patients demonstrated that active disease was associated with an increased frequency of mature neutrophils and a decreased frequency of monocytes, in particular intermediate monocytes. Moreover, GPA patients with a tendency to relapse displayed an increased frequency of mature neutrophils with increased expression of CD177^+^. Relapsing MPA patients, on the other hand, showed decreased frequency of intermediate monocytes. Finally, rituximab treatment was associated with increased frequencies of classical and intermediate monocytes. In conclusion, AAV patients exhibit a skewing of different neutrophil and monocyte subpopulations that are associated with disease subtypes, disease activity, rituximab treatment, and propensity to relapse. These changes may contribute to the inflammatory process and could potentially be used as biomarkers for relapse prediction.

## 1. Introduction

Antineutrophil cytoplasmic antibody (ANCA)-associated vasculitis (AAV) is a group of autoimmune diseases with severe inflammation affecting small blood vessels, leading to extravascular inflammation, tissue damage, fibrosis, and eventually loss of function [1]. The upper and lower respiratory tract and kidneys are most commonly and often severely affected, but any organ could be involved. Autoantibodies to leukocytes, the ANCAs are thought to be important in AAV, both by direct attack and as markers of specific inflammation. The two major autoantigens recognized by ANCAs are proteinase 3 (PR3) and myeloperoxidase (MPO), mainly found in the primary granules of neutrophils and in the lysosomes of monocytes. AAV can be divided into three main subtypes based on serology and clinical features: granulomatosis with polyangiitis (GPA), microscopic polyangiitis (MPA), and eosinophilic GPA (EGPA). An alternative classification based on ANCA-subtype has also been suggested [2].

The incidence has increased with an estimated worldwide peak to 28.3 per million population per year regarding GPA during 2005 and to 15.2 per million population per year regarding MPA during 2008 [3]. A meta-analysis covering 4,547 patients with AAV found that the occurrence varies within geographic areas, where GPA and MPA display higher prevalence rates in the northern hemisphere compared to the southern hemisphere [4]. GPA is more common in Northern Europe and Australia/New Zealand, while MPA predominates in Asia and Southern Europe [5]. The etiology of AAV is not completely known, and risk factors such as genetic predisposition, infectious disease, and environmental factors are involved in the pathogenesis, resulting in loss of immunological tolerance to PR3 or MPO [1].

The neutrophils are thought to play an important role in the pathogenesis of AAV and are found in and around inflamed vessel walls. ANCAs activate neutrophils, leading to degranulation and production of reactive oxygen species, complement activation, and release of neutrophil extracellular traps [6, 7]. Neutrophils cause early distinctive ultrastructural lesions in MPA patients by attachment of vascular endothelial cells, migration to the extravascular space and release of neutrophil components [8]. Mature segment nucleated neutrophils express CD10^+^CD16^high^, whereas cells expressing CD10^−^CD16^dim^ in peripheral blood are thought to reflect an increased mobilization of neutrophils from the bone marrow. We and others have previously shown that AAV patients display increased frequencies of both circulating CD10^−^CD16^dim^ and mature CD10^+^CD16^high^ neutrophils, suggesting a combination of increased bone marrow release and prolonged survival of neutrophils [9]. CD177 is exclusively expressed in a subset of neutrophils and forms a high-affinity complex with PR3 [10]. The function of CD177 is unclear; however, there have been several reports demonstrating a correlation between a large CD177^+^/mPR3^high^ neutrophil population and the occurrence of AAV.

The role of monocytes in AAV has been studied less and not fully established. However, monocytes and macrophages are frequently found in the vascular infiltrates of affected organs such as kidneys and lungs of patients with AAV. Moreover, monocytes are recruited to AAV lesions and mature in the tissue into macrophages. High numbers of infiltrating macrophages are, for example, seen already in early lesions of ANCA-associated glomerulonephritis [11]. Monocytes can be subdivided into three subsets based on their expression of the lipopolysaccharide receptor, CD14, and the Fc*γ*RIII, CD16 [12]. The classical (CD14^++^CD16^−^) monocytes compromise the largest monocyte subset and have a high phagocytic capacity, proinflammatory properties and antimicrobial effects [13]. Nonclassical (CD14^−^CD16^+^) monocytes can detect viruses and nucleic acids and are thought to be patrolling caretakers of arterial vessels, important for maintaining endothelial homeostasis [14] and in search of injury [13]. The intermediate monocytes (CD14^++^CD16^+^) have been implicated in cytokine production and antigen presentation, proliferation and stimulation of T cells, inflammatory responses, and in angiogenesis [13, 15]. The frequencies of circulating monocyte subsets in healthy individuals are about 85% classical, 5% intermediate, and 10% nonclassical [16]. We hypothesize that the innate immune system is of importance to maintain the chronic inflammation in AAV. The aim of this study was to characterize granulocyte and monocyte subsets in a large cohort of AAV patients with emphasis on disease activity and tendency to relapse.

## 2. Materials and Methods

### 2.1. Patients and Controls

AAV patients were recruited to this retrospective study at the time of diagnosis or when attending clinical routine visits at the outpatient clinic of Nephrology or Rheumatology, Skåne University Hospital, Lund, Sweden, between 2011 and 2020. One hundred thirty-eight patients diagnosed with GPA or MPA between 1978 and 2018 were consecutively enrolled. One hundred five patients fulfilled the inclusion criteria. Exclusion criteria included ongoing infection, malignancy, other autoimmune diseases, dialysis/plasmapheresis treatment, transplantation, other diseases affecting the clinical evaluation, and less than 500 monocytes in the flow cytometry analysis. Patients were classified to GPA or MPA according to the consensus methodology described by Watts et al. [17] in 2007. Patients with symptoms and signs that are characteristic or compatible with AAV and no other diagnosis to account for the symptoms/signs were entered into the classification algorithm. If they fulfilled the ACR (American College of Rheumatology) or Lanham criteria for EGPA, they were diagnosed as EGPA and excluded from the study. To be included into the GPA diagnosis, at least one of the following criteria must be fulfilled: (i) ACR criteria for GPA, (ii) histology compatible with GPA, (iii) histology compatible with MPA and GPA surrogate markers, (iv) no histology but GPA surrogate markers and positive MPO- or PR3-ANCA. Patients not fulfilling any of these criteria were diagnosed as MPA if they fulfilled at least one of the following criteria: (i) clinical features and histology compatible with small vessel vasculitis and no GPA surrogate markers, or (ii) no histology, no GPA surrogate markers, but surrogate markers for renal vasculitis and positive MPO- or PR3-ANCA.

The patient cohort consisted of 68 patients with GPA and 37 patients with MPA. Eighty-five patients were sampled more than once. For cross-sectional analysis, the last blood sample of each patient (*n* = 105) was used. Twenty-three patients had at least one relapse during the sampling period. Most of the patients were on treatment at the time of sampling, and 26 had no treatment (Table 1). One hundred twenty-six healthy blood donors (healthy controls (HCs)) at the Blood Donor Center in Lund, aged 21–72, were recruited as controls. No other laboratory or clinical parameters for HC were retrieved.

The study was approved by the regional ethical review board in Lund, Sweden (permit number 2008/110 and 2021-04168). Prior to inclusion, all subjects gave written informed consent.

### 2.2. Laboratory and Clinical Parameters

Disease activity was assessed using the Birmingham Vasculitis Activity Score version 3 (BVAS3) [18]. Clinical and demographic characteristics are reported in Table 1. Active disease was defined as BVAS3 ≥ 2 and remission as BVAS3 ≤ 1 when standard of care criteria were fulfilled. Tendency to relapse (Ttr) was defined as the recurrence of disease after complete remission when the patient had received standard of care and completed at least 1 year of follow-up. In this setting, recurrence of disease was defined as BVAS3 > 1 and an increased dose of immunosuppressive treatment.

ANCA specificity was determined with ELISA at Wieslab AB, Malmö, or at the Department of Clinical Immunology and Transfusion Medicine, Region Skåne, Lund. Other laboratory data, including white blood cell (WBC) count, C-reactive protein (CRP), and creatinine, were analyzed as routine clinical samples at the Department of Clinical Chemistry, Region Skåne, Lund. eGFR (estimated glomerular filtration rate) was calculated by the CKD-EPI Creatinine (2009) equation (http://www.mdrd.com).

### 2.3. Phenotypic Characterization of Granulocytes and Monocytes

Basophils, eosinophils, neutrophils, and monocytes were included in all experiments and analyses of this study. Heparinized peripheral blood (stored at room temperature for less than 24 hr and protected from light) was lysed using 0.84% ammonium chloride. The leukocytes were stained for surface expression of various granulocyte and monocyte markers and processed using a FACSCanto II (Becton Dickinson, BD, New York, USA) with FACSDiva software for data collection, and analyzed with Kaluza software version 2.1 (Beckman Coulter, Brea, CA, USA). At least 40,000 granulocytes were acquired based on forward and side scatter properties. The gating strategy is illustrated in Figure S1. A few changes regarding antibody clone or fluorophore were made after careful evaluation during the sample collection period. Antibodies recognizing the following antigens (clone) were used: CD14 (M5E2) BD Bioscience, California, USA or (HCD14) Biolegend, California, USA. CD10 (HI10a), CD16 (HIB19 or 3G8) and CD193 (5E8) BD Bioscience. CD177 (MEM-166 or GO25H7), Siglec-8 (7C9), and CD88 (S5/1) Biolegend. The CD88 antibody was included in the panel but not analyzed in this study. The addition of CD88 antibody did not interfere with the analysis.

### 2.4. Statistical Analysis

Statistical analyses were performed with GraphPad Prism 9.0.1 software (GraphPad Software, San Diego, CA, USA). Mann–Whitney *U* test was used for two-group comparisons, and the Wilcoxon signed-rank test was used to compare paired samples. Values are expressed as median with interquartile range (IQR) or range. Results were considered statistically significant at *p* < 0.05.

## 3. Results

### 3.1. Patient Characteristics

Patient characteristics and demographics at the time of the last sampling are reported in Table 1. About two-thirds were diagnosed with GPA and one-third with MPA. The female-to-male ratio was 1 : 1.1, and the median age at the time of sampling was 69 years (56–77). Fifty-seven percent were PR3-ANCA positive, 38% were MPO-ANCA positive, and 3% were ANCA negative. Most of the patients were in remission. Thirteen percent displayed disease activity according to BVAS3, with a median score of 5 (range 2–16). About half of the patients (45%) showed a tendency to relapse, with the highest prevalence in the GPA group.

The Ttr status could not be determined for 24 patients due to a short follow-up period or lack of clinical information. The pharmacological treatment at the time of sampling varied (Table 1). Twenty-six patients were unmedicated.

### 3.2. Increased Frequency of Neutrophils and Intermediate Monocytes in AAV

**Figure 1:**
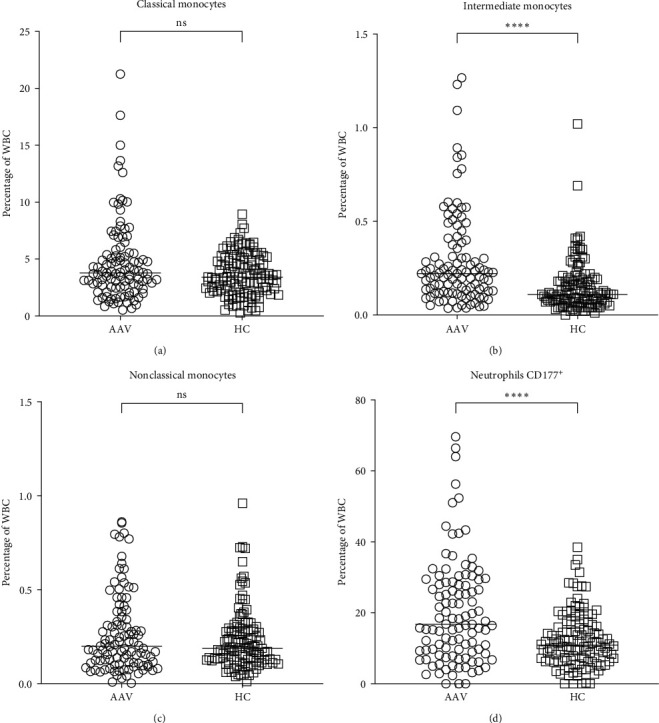
Increased frequency of intermediate monocytes in AAV patients compared to healthy controls. The frequencies of (a) classical (CD14^++^CD16^−^), (b) intermediate (CD14^++^CD16^+^), (c) nonclassical (CD14^−^CD16^+^) monocytes, and (d) neutrophils CD177^+^ in AAV patients and HC, analyzed with flow cytometry, as described in Section 2. AAV patients present a higher frequency of intermediate monocytes and neutrophils CD177^+^, but not of classical and nonclassical, compared to HC. Mann–Whitney *U* test was used to calculate the level of significance. Data are presented with medians.  ^*∗∗∗∗*^Indicates *p*-value < 0.0001. WBC, white blood cell; AAV, antineutrophil cytoplasmic antibody (ANCA)-associated vasculitis; HC, healthy controls; ns, not significant.

Neutrophils are, in general, the first cells recruited to an inflammatory site [19] and are followed by monocytes that migrate into the tissue and become mature macrophages. In peripheral blood from AAV patients, there were increased frequencies of early released CD16^dim^ neutrophils (*p*=0.004), mature CD16^high^ neutrophils (*p* < 0.0001) and CD177^+^ neutrophils (*p* < 0.0001) (Table 2 and Figure 1), compared to HC. No significant difference in eosinophils or basophils was observed. These results are in *p* < 0.0001 line with our previous study [10], indicating that AAV patients have skewed neutrophil and monocyte profiles (Table 2).

To further study the monocyte population, classical, intermediate, and nonclassical monocytes were defined based on their surface expression of CD14 and CD16. AAV patients displayed an increased frequency of intermediate monocytes of WBC (*p* < 0.0001, Figure 1) and of total monocytes (*p*=0.04, data not shown) compared to HC. There were no statistically significant differences in classical and nonclassical monocyte frequencies between AAV patients and HC.

In order to examine if the clinical disease subtype had an impact on varying leukocyte phenotypes, we compared the MPA and GPA cohorts to HCs. The findings revealed a comparable pattern in neutrophil phenotypes as observed in the overall disease cohort (Tables S1 and S2). Interestingly, the MPA patients only showed a significant change in the frequency of intermediate monocytes (Tables S1 and S2).

### 3.3. Decreased Frequency of Classical Monocytes in MPA Compared to GPA Patients

**Figure 2:**
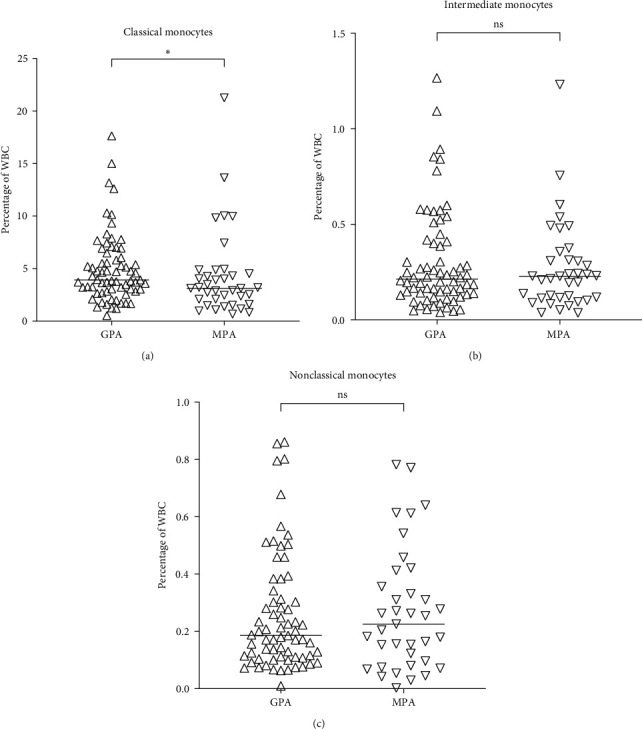
Decreased frequency of classical monocytes in MPA compared to GPA patients. The frequencies of (a) classical (CD14^++^CD16^−^), (b) intermediate (CD14^++^CD16^+^), and (c) nonclassical (CD14^−^CD16^+^) monocytes in GPA and MPA patients. Analysis of monocytes in MPA patients compared to GPA patients evidenced a lower frequency of classical monocytes. No significant difference was shown between the two groups regarding intermediate and nonclassical monocytes. The Mann–Whitney *U* test was used to calculate the level of significance. Data are presented with medians.  ^*∗*^Indicates *p*-value < 0.01. WBC, white blood cell; GPA, granulomatosis with polyangiitis; MPA, microscopic polyangiitis, ns, not significant.

To further investigate whether the AAV subtypes were associated with specific changes in the distribution of neutrophils and monocytes, the patient cohort was divided by diagnosis or ANCA serotype in a cross-sectional analysis of the last sample for each patient. Basophils, eosinophils, neutrophils, and monocytes were included in the analysis. The only difference noted was a decreased frequency of classical monocytes in MPA as compared to GPA patients (*p*=0.04, Figure 2 and Table S3). No significant difference in the frequencies of the investigated cell types was found when patients were divided by ANCA serotype (Table S4).

### 3.4. Granulocytes and Monocytes in Active Disease and Remission

**Figure 3:**
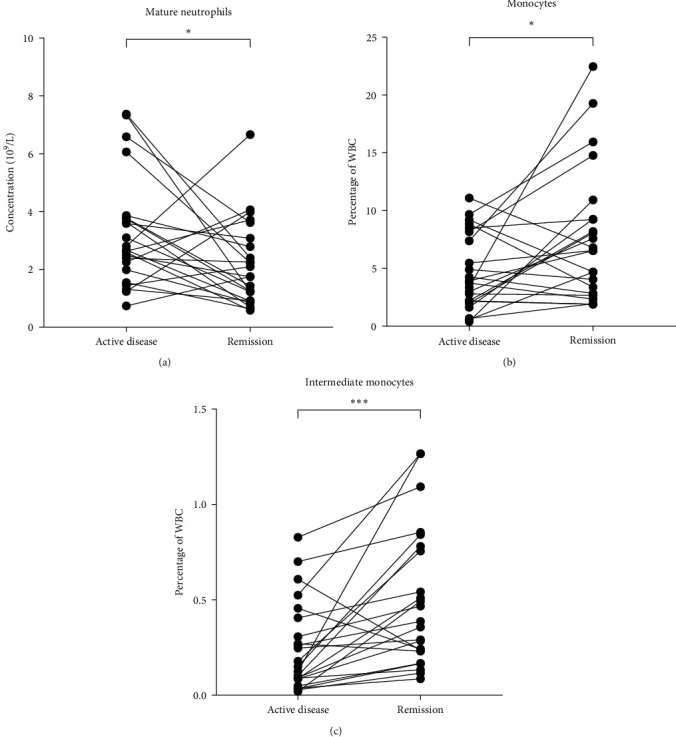
Concentration of (a) mature (CD16^high^) neutrophils, and frequencies of (b) total monocytes, and (c) intermediate (CD14^++^CD16^+^) monocytes in 23 patients with antineutrophil cytoplasmic antibody (ANCA)-associated vasculitis (AAV) in active disease and remission. Patients with active disease present higher concentrations of mature neutrophils but lower frequencies of total monocytes and intermediate monocytes compared to patients in remission. Wilcoxon matched-pairs signed rank test was used to calculate the level of significance. ^*∗*^and  ^*∗∗∗*^Indicate *p*-value < 0.05 and <0.001, respectively. WBC, white blood cell.

To determine frequencies of granulocytes in relation to disease activity, 23 patients who had been sampled repeatedly, with at least one blood sample during the active disease period and one during remission, were defined. The analysis included the last collected sample and the one prior to that from either remission or active disease, depending on the disease activity of the last sample. No specific time interval between the two samples was considered. We found that the concentration of mature CD16^high^ neutrophils was increased (*p*=0.04), and the frequencies of total (*p*=0.04) and intermediate (*p*=0.001) monocytes were decreased in active disease (Figure 3). This could be related to the recruitment of monocytes to the site of inflammation. A similar phenomenon was observed in MPA patients with a tendency to relapse (see below), indicating that they have an ongoing low-grade inflammation.

Moreover, patients in remission or with active disease were compared to HC to study whether the frequency of different cell types changed due to disease activity. For the comparison to the HC group, the last blood sample was used.

The percentage of the various cell types showed a similar pattern for patients in remission and in active disease when compared to HC (Tables S5 and S6). However, for the patients with active disease only, the frequency of neutrophils CD177^+^ reached statistical significance. This might be due to the low number of patients (*n* = 14) in this group compared to the HC (*n* = 126) and the remission group (*n* = 91) (Tables S5 and S6).

### 3.5. The Cell Distribution in Relapsing Patients Differed between GPA and MPA

**Figure 4:**
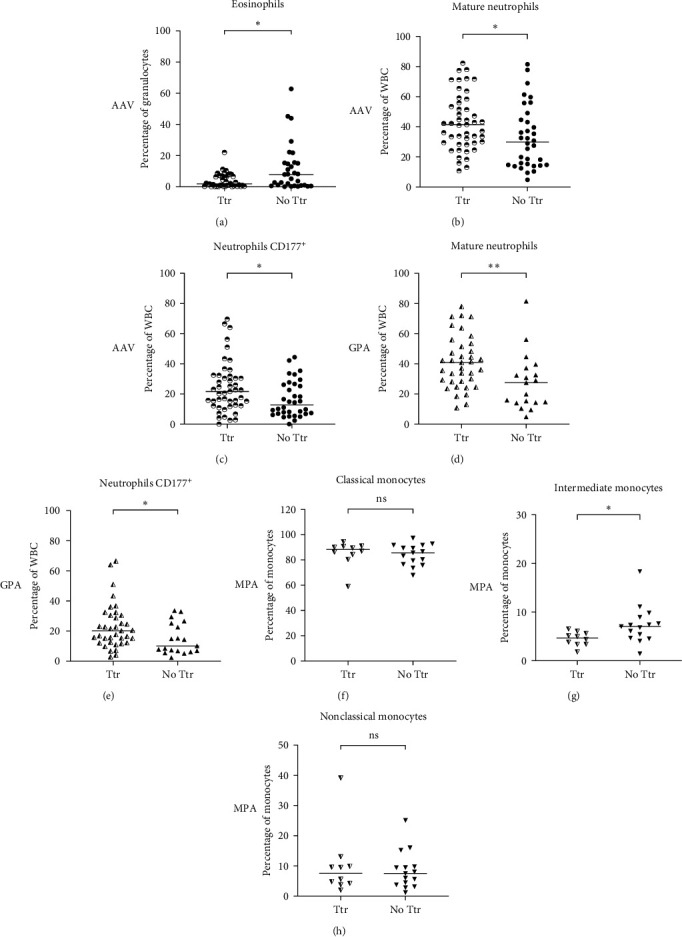
Comparison of granulocyte and monocyte frequencies in patients with and without a tendency to relapse (Ttr). Frequencies of (a) eosinophils, (b) mature CD16^high^, and (c) CD177^+^ neutrophils in AAV patients with Ttr or No Ttr, where patients with Ttr presented decreased frequency of eosinophils, but increased frequency of mature neutrophils and CD177^+^ neutrophils. Analysis of cell subsets in GPA patients with Ttr and without Ttr demonstrated statistically significant increased frequencies of (d) mature CD16^high^ and (e) CD177^+^ neutrophils. The investigation of the cell subsets in MPA patients with Ttr and without Ttr identified decreased frequency of (g) intermediate (CD14^++^CD16^+^) monocytes but no statistically significant difference of (f) classical (CD14^++^CD16^−^), and (h) nonclassical (CD14^−^CD16^+^) monocytes. Mann–Whitney *U* test was used to calculate the level of significance. Data are presented with medians.  ^*∗*^and  ^*∗∗*^Indicate *p*-value < 0.05 and <0.01, respectively. AAV, antineutrophil cytoplasmic antibody (ANCA)-associated vasculitis; Ttr, tendency to relapse; No Ttr, no tendency to relapse; GPA, granulomatosis with polyangiitis; MPA, microscopic polyangiitis; ns, not significant.

Patients diagnosed with AAV have at least one active disease period, often at the time of diagnosis, followed by remission induced by treatment. Unfortunately, long-term remission is not always possible to achieve, and two or more active disease periods are frequently observed between remission intervals, defining a tendency to relapse (Ttr). AAV patients who fulfilled the criteria for evaluation of relapse tendency were divided into two groups: the Ttr group (*n* = 47) and the No Ttr group (*n* = 34). Ttr patients showed decreased frequency of eosinophils (*p*=0.02) and increased frequency of mature CD16^high^ neutrophils (*p*=0.02) (Figures 4(a) and 4(b)). In addition, a high frequency of CD177^+^ neutrophils (*p*=0.03) were observed (Figure 4(c)). No differences in monocyte frequencies were observed.

When dividing the patients based on disease phenotype, GPA patients with Ttr (*n* = 37) displayed higher frequencies of mature and CD177^+^ neutrophils, whilst MPA patients with Ttr (*n* = 10) had decreased frequency of intermediate monocytes (Figure 4(d)–4(h)). These results indicate specific differences in neutrophil and monocyte populations between GPA and MPA patients regarding relapse tendency.

### 3.6. Rituximab Treatment Was Associated with Increased Frequencies of Classical and Intermediate Monocytes

**Figure 5:**
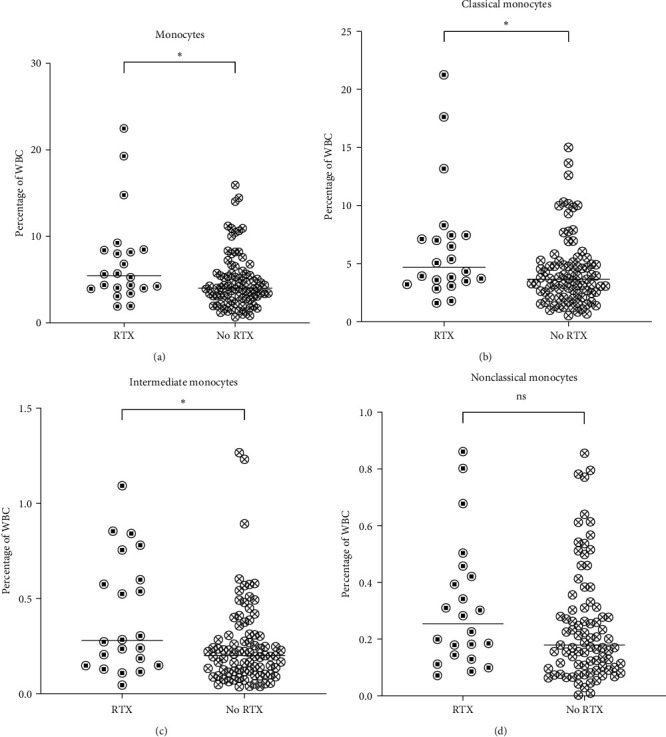
Increased monocyte frequencies in antineutrophil cytoplasmic antibody (ANCA)-associated vasculitis (AAV) patients with rituximab treatment during the past 365 days. The frequencies of (a) total monocytes, (b) classical (CD14^++^CD16^−^), (c) intermediate (CD14^++^CD16^+^), and (d) nonclassical (CD14^−^CD16^+^) monocytes in patients with RTX or No RTX treatment. Total, classical, and intermediate monocytes were presented with higher frequency in patients in RTX treatment. No significant difference was observed in the comparison of nonclassical monocytes between the two groups. Mann–Whitney *U* test was used to calculate the level of significance. Data are presented with medians.  ^*∗*^Indicates *p*-value < 0.05. WBC, white blood cell; RTX, rituximab treatment; No RTX, no rituximab treatment; ns, not significant.

Rituximab (RTX) is used in modern AAV therapy for the initial remission induction and for remission maintenance. RTX is an anti-CD20 monoclonal B-cell depleting antibody used in several diseases and is known to affect the circulating pool of neutrophils in some patients [20]. To investigate if RTX treatment was associated with changes in neutrophil and monocyte populations, AAV patients who had received at least one RTX treatment during the past year from the date of sampling (*n* = 22) were compared to patients without RTX (*n* = 83). RTX-treated patients displayed a higher frequency of total monocytes (*p*=0.03), and more specifically, of classical (*p*=0.03) and intermediate (*p*=0.03) monocytes (Figure 5). Similar results were obtained when analyzing the concentration (10^9^/L) of monocytes and monocyte subsets in these groups (data not shown), indicating that there is an absolute increase of classical and intermediate monocytes in RTX-treated patients.

No differences in the granulocyte subsets with respect to RTX treatment were observed (Table S7).

## 4. Discussion

Neutrophils and monocytes play important roles in AAV pathogenesis, both directly as effector cells and indirectly as regulators of the adaptive immune response. Most research on AAV pathogenesis has focused on neutrophils, even though the ANCA stimulatory effect on monocytes was described at the same time [21]. However, during the last decade, a new interest for monocytes has evolved, and several inflammatory diseases have been reported to be associated with an increased population of intermediate monocytes, including AAV, other kidney diseases, rheumatoid arthritis, and asthma. However, there is no consensus regarding the association with disease activity [22−25]. Here, we report that AAV patients exhibit a changed repertoire of neutrophil and monocyte subpopulations that are associated with disease subtype, activity, rituximab treatment, and tendency to relapse.

The association between monocytes and disease activity has been debated. In this study, AAV patients, in general, displayed an increased frequency of monocytes and the intermediate monocyte subset compared with healthy individuals. However, patients with an active disease showed lower frequencies of monocyte and intermediate monocytes than patients in remission, which might reflect that monocytes are recruited to the site of inflammation. A similar phenomenon was observed in MPA patients with a tendency to relapse, indicating that they have an ongoing low-grade inflammation. These findings are in line with studies on lupus patients, where intermediate monocytes have been reported to take part in the local inflammation by enhanced differentiation to CD16^+^ tissue macrophages [26]. Moreover, intermediate monocytes primed with TNF-*α* in vitro expressed increased levels of IL-1*β*, IL-6, and IL-8 after activation with anti-MPO antibodies [27] that contributed to the inflammatory process.

The increase in intermediate monocytes in the AAV patients at large is coherent with other studies; however, most reports do not observe any changes in the total monocyte frequency [27, 28]. This might be explained by different study designs, the number of included patients, and their propensity to relapse. Moreover, the treatment regime might be an additional factor influencing the monocyte subsets.

Neutrophils have been extensively studied in AAV, and here we could confirm previous results showing that AAV patients have an increased frequency of CD177^+^ neutrophils [10] and that the neutrophil counts are increased during active disease. CD177 is coexpressed with PR3 on the surface of neutrophils [9] and has been associated with AAV, increased disease activity, and poor clinical outcome [29−31]. Our results support these findings and could also show that GPA patients with a tendency to relapse have an increased proportion of neutrophils expressing CD177. Interestingly, no such correlation was found in the MPA group, indicating a difference in the pathogenesis between MPA and GPA that is reflected by the expansion of different immune cells. Further support for this theory is the finding that GPA patients had an increased frequency of classical (CD14^++^CD16^−^) inflammatory monocytes that could not be explained in increased disease activity (BVAS) or inflammatory activity (CRP) (Table 1).

Previous studies of AAV patients have reported genetic associations with ANCA subtype [2], where PR3-ANCA was associated with HLA-DP and the genes encoding *α* (1)-antitrypsin and PR3, and MPO-ANCA with HLA-DQ. In this study, we could not identify any differences regarding the ANCA subtype, suggesting that clinical phenotype is more dependent on underlying chronic inflammatory mechanisms. Future studies might be able to validate this difference.

In this study, we show that RTX treatment might affect the various monocyte subpopulations. Patients who received RTX displayed higher frequencies of both classical and intermediate monocytes. Sustained remission due to RTX has been reported in almost 90% of refractory patients. Different study trials, such as RITUXVAS and RAVE [32, 33], have concluded that RTX and glucocorticoid therapy are superior to the standard treatment for induction of remission in relapsing patients. The mechanism of action of RTX is still not completely understood, but it leads to cytotoxic cell killing or apoptosis of B cells, and the level of B-cell depletion is positively related to the RTX levels [32]. Monocytes/macrophages take an active part in this antibody-dependent cell cytotoxicity of B cells, an action that might change their phenotype. Moreover, RTX has been described to change the cytokine profile in patients, including cytokines such as B-cell activating factor, interleukin (IL)-10, and IL-15 [34, 35], which could affect monocyte differentiation and phenotype. Additionally, studies are needed to further study the RTX effect on monocytes and macrophages.

## 5. Conclusions

In the present study, we report that AAV patients exhibit a skewing of neutrophil and monocyte subpopulations that are associated with clinical disease subtype, disease activity, and tendency to relapse. There were no differences associated with the ANCA subtype. Our data rather suggest that clinical features might be more dependent on underlying chronic inflammatory mechanisms, reflected in the expansion of different cell subsets, than previously reported genetic factors. Moreover, rituximab treatment was associated with changes in the monocyte population, and further investigations are needed to evaluate the association with treatment response. Finally, the identified changes in neutrophil and monocyte subsets could potentially be used as biomarkers for relapse prediction.

## Figures and Tables

**Table 1:** Patient characteristics and demographics.

	AAV (*n* = 105)	GPA (*n* = 68)	MPA (*n* = 37)
Age, (year), median (IQR)	69 (56–77)	65.5 (48–74)	73 (63–80)
Gender, female/male, *n* (%)	50 (48)/55 (52)	30 (44)/38 (56)	20 (54)/17 (46)
Age at diagnosis, years, median (IQR)	59 (39–70)	52 (34–66)	68 (54–74)
ANCA specificity, *n* (%)			
PR3	60 (57)	54 (79)	6 (16)
MPO	40 (38)	12 (18)	28 (76)
PR3 and MPO	1 (1)	0 (0)	1 (3)
No ANCA	3 (3)	2 (3)	1 (3)
Data not available	1 (1)	0 (0)	1 (3)
Disease activity			
Active disease, *n* (%)	14 (13)	8 (12)	6 (16)
BVAS3, median (IQR)	5 (3–13)	3 (2–6)	14 (5–15)
Remission, *n* (%)	91 (87)	60 (88)	31 (84)
Tendency to relapse, *n* (%)			
Yes	47 (45)	37 (54)	10 (27)
No	34 (32)	19 (28)	15 (41)
Not applicable	24 (23)	12 (18)	12 (32)
WBC, ×10^9^/L, median (IQR)	6.5 (5.2–8.1)	6.0 (5.1–7.9)	6.8 (5.5–8.8)
P-CRP, (mg/L), median (IQR)	2.3 (1.2–5.6)	2.0 (1.1–4.0)	4.7 (1.4–8.5)
S-creatinine, (*μ*mol/L), median (IQR)	110 (82–154)	98 (78–144)	133 (97–180)
eGFR, (mL/min/1.73 m^2^), median (IQR)	51 (37–74)	60 (40–84)	39 (27–56)
Medication, *n* (%), dose, median (IQR)			
Rituximab	22 (21)	18 (26)	4 (11)
Prednisolone,	60 (57)	36 (53)	24 (65)
mg/day	5 (5–10)	5 (5–7.5)	10 (5–14)
Azathioprine,	30 (29)	18 (26)	12 (32)
mg/day	100 (69–100)	100 (69–150)	75 (56–100)
Methotrexate,	6 (9)	6 (9)	0 (0)
mg/week	23 (20–25)	23 (20–25)	
Mycophenolate mofetil,	7 (7)	5 (7)	2 (5)
mg/day	1.5 (1.5–2)	2 (1–2)	1.5 (1.5)
Cyclophosphamide	8 (8)	3 (4)	5 (14)
No medication	26 (25)	18 (26)	8 (22)

AAV, antineutrophil cytoplasmic antibody (ANCA)-associated vasculitis; GPA, granulomatosis with polyangiitis; MPA, microscopic polyangiitis; IQR, interquartile range; ANCA, antineutrophil cytoplasmatic antibody; PR3, proteinase 3; MPO, myeloperoxidase; BVAS3, Birmingham vasculitis activity score version 3; WBC, white blood cell; CRP, C-reactive protein. eGFR (estimated glomerular filtration rate) was calculated by the CKD-EPI Creatinine (2009) equation (http://www.mdrd.com). Reference range adults: WBC 3.5–8.8 × 10^9^/L, CRP < 5.0 mg/L, creatinine 60–105 *µ*mol/L (male), 45–90 *µ*mol/L (female).

**Table 2:** Leucocytes frequency in ANCA-associated vasculitis patients and healthy controls.

Cell type (% of WBC)	AAV patients (*n* = 105)	HC (*n* = 126)	*p*-Value
Basophils	0.8 (0.03–7.2)	0.7 (<0.001–2.5)	ns
Eosinophils	1.9 (0.02–20)	2.2 (0.2–16)	ns
Neutrophils CD16^dim^	2.1 (0.06–16)	1.5 (0.008–10)	0.004
Mature neutrophils	36 (4.8–82)	25 (3.0–63)	<0.0001
Neutrophils CD177^+^	17 (0–70)	11 (0–39)	<0.0001
Monocytes, total	4.2 (0.7–23)	3.8 (0.3–9.2)	0.04
Classical monocytes	3.8 (0.5–21.3)	3.4 (0.3–9)	ns
Intermediate monocytes	0.2 (0.04–1.3)	0.1 (0–1.02)	<0.0001
Nonclassical monocytes	0.2 (<0.1–0.9)	0.2 (0.01–1)	ns

Mann–Whitney *U* test was used to calculate the level of significance. Data are presented with medians (ranges). For the basophil analysis, 94 AAV patients and 67 HC were included. For eosinophil analysis, 92 AAV patients and 83 HC were included. AAV, antineutrophil cytoplasmic antibody (ANCA)-associated vasculitis; HC, healthy controls; ns, not significant.

## Data Availability

Raw data files from flow cytometry datasets used in the current study are available from the corresponding author upon reasonable request.

## Additional Points

*Limitations*. Limitations of this study include heterogeneity of disease duration and lack of timing of blood collections (samples were obtained from patients at clinical routine visits). Controls were not age- or gender-matched. An additional important limitation is the possible influence of medications on disease severity and the immune cell distribution.

## Supplementary Materials

Supplementary Materials Figure S1: gating strategy for granulocyte and monocyte analysis. Table S1: leukocytes frequency in MPA patients and healthy controls. Table S2: leukocytes frequency in GPA patients and healthy controls. Table S3: comparison of leukocytes in GPA and MPA patients. Table S4: comparison of leukocytes in MPO and PR3-positive AAV patients. Table S5: leukocytes frequency in patients in active disease and healthy controls. Table S6: leukocytes frequency in patients in remission and healthy controls. Table S7: comparison of leucocytes in AAV patients with and without RTX treatment.

## References

[1] Kitching A. R., Anders H.-J., Basu N. (2020). ANCA-associated vasculitis. *Nature Reviews Disease Primers*.

[2] Lyons P. A., Rayner T. F., Trivedi S. (2012). Genetically distinct subsets within ANCA-associated vasculitis. *New England Journal of Medicine*.

[3] Watts R. A., Mooney J., Skinner J., Scott D. G. I., MacGregor A. J. (2012). The contrasting epidemiology of granulomatosis with polyangiitis (Wegener’s) and microscopic polyangiitis. *Rheumatology*.

[4] Redondo-Rodriguez R., Mena-Vázquez N., Cabezas-Lucena A. M., Manrique-Arija S., Mucientes A., Fernández-Nebro A. (2022). Systematic review and metaanalysis of worldwide incidence and prevalence of antineutrophil cytoplasmic antibody (ANCA) associated vasculitis. *Journal of Clinical Medicine*.

[5] Geetha D., Jefferson J. A. (2020). ANCA-associated vasculitis: core curriculum 2020. *American Journal of Kidney Diseases*.

[6] Xiao H., Schreiber A., Heeringa P., Falk R. J., Jennette J. C. (2007). Alternative complement pathway in the pathogenesis of disease mediated by anti-neutrophil cytoplasmic autoantibodies. *The American Journal of Pathology*.

[7] Kessenbrock K., Krumbholz M., Schönermarck U. (2009). Netting neutrophils in autoimmune small-vessel vasculitis. *Nature Medicine*.

[8] Koike H., Furukawa S., Mouri N., Fukami Y., Iijima M., Katsuno M. (2022). Early ultrastructural lesions of anti-neutrophil cytoplasmic antibody- versus complement-associated vasculitis. *Neuropathology*.

[9] Abdgawad M., Gunnarsson L., Bengtsson A. A. (2010). Elevated neutrophil membrane expression of proteinase 3 is dependent upon CD177 expression. *Clinical and Experimental Immunology*.

[10] Johansson A. C., Ohlsson S., Pettersson A. (2016). Impaired phagocytosis and reactive oxygen species production in phagocytes is associated with systemic vasculitis. *Arthritis Research & Therapy*.

[11] Zhao L., David M. Z., Hyjek E., Chang A., Meehan S. M. (2015). M2 macrophage infiltrates in the early stages of ANCA-associated pauci-immune necrotizing GN. *Clinical Journal of the American Society of Nephrology*.

[12] Ziegler-Heitbrock L., Ancuta P., Crowe S. (2010). Nomenclature of monocytes and dendritic cells in blood. *Blood*.

[13] Sampath P., Moideen K., Ranganathan U. D., Bethunaickan R. (2018). Monocyte subsets: phenotypes and function in tuberculosis infection. *Frontiers in Immunology*.

[14] Thomas G., Tacke R., Hedrick C. C., Hanna R. N. (2015). Nonclassical patrolling monocyte function in the vasculature. *Arteriosclerosis, Thrombosis, and Vascular Biology*.

[15] Wong K. L., Yeap W. H., Tai J. J. Y., Ong S. M., Dang T. M., Wong S. C. (2012). The three human monocyte subsets: implications for health and disease. *Immunologic Research*.

[16] Ravenhill B. J., Soday L., Houghton J., Antrobus R., Weekes M. P. (2020). Comprehensive cell surface proteomics defines markers of classical, intermediate and non-classical monocytes. *Scientific Reports*.

[17] Watts R., Lane S., Hanslik T. (2007). Development and validation of a consensus methodology for the classification of the ANCA-associated vasculitides and polyarteritis nodosa for epidemiological studies. *Annals of the Rheumatic Diseases*.

[18] Mukhtyar C., Lee R., Brown D. (2009). Modification and validation of the Birmingham vasculitis activity score (version 3). *Annals of the Rheumatic Diseases*.

[19] Aristizábal B., González A., Anaya J. M., Shoenfeld Y., Rojas-Villarraga A., Levy R. A., Cervera R. Innate immune system. *Autoimmunity: from Bench to Bedside*.

[20] Kronbichler A., Windpessl M., Pieringer H., Jayne D. R. W. (2017). Rituximab for immunologic renal disease: what the nephrologist needs to know. *Autoimmunity Reviews*.

[21] Charles L. A., Falk R. J., Jennette J. C. (1992). Reactivity of antineutrophil cytoplasmic autoantibodies with mononuclear phagocytes. *Journal of Leukocyte Biology*.

[22] Vegting Y., Vogt L., Anders H. J., de Winther M. P. J., Bemelman F. J., Hilhorst M. L. (2021). Monocytes and macrophages in ANCA-associated vasculitis. *Autoimmunity Reviews*.

[23] Rossol M., Kraus S., Pierer M., Baerwald C., Wagner U. (2012). The CD14^bright^CD16+ monocyte subset is expanded in rheumatoid arthritis and promotes expansion of the Th17 cell population. *Arthritis and Rheumatism*.

[24] Heron M., Grutters J. C., van Velzen-Blad H., Veltkamp M., Claessen A. M. E., van den Bosch J. M. M. (2008). Increased expression of CD16, CD69, and very late antigen-1 on blood monocytes in active sarcoidosis. *Chest*.

[25] Moniuszko M., Bodzenta-Lukaszyk A., Kowal K., Lenczewska D., Dabrowska M. (2009). Enhanced frequencies of CD14++CD16+, but not CD14+CD16+, peripheral blood monocytes in severe asthmatic patients. *Clinical Immunology*.

[26] Arazi A., Rao D. A., Berthier C. C. (2019). The immune cell landscape in kidneys of patients with lupus nephritis. *Nature Immunology*.

[27] O’Brien Eóin C., Abdulahad W. H., Rutgers A. (2015). Intermediate monocytes in ANCA vasculitis: increased surface expression of ANCA autoantigens and IL-1*β* secretion in response to anti-MPO antibodies. *Scientific Reports*.

[28] Matsumoto K., Suzuki K., Yoshimoto K. (2020). Longitudinal immune cell monitoring identified CD14++ CD16+ intermediate monocyte as a marker of relapse in patients with ANCA-associated vasculitis. *Arthritis Research & Therapy*.

[29] Rarok A. A., Stegeman C. A., Limburg P. C., Kallenberg C. G. M. (2002). Neutrophil membrane expression of proteinase 3 (PR3) is related to relapse in PR3-ANCA-associated vasculitis. *Journal of The American Society of Nephrology*.

[30] Schreiber A., Otto B., Ju X. (2005). Membrane proteinase 3 expression in patients with Wegener’s granulomatosis and in human hematopoietic stem cell-derived neutrophils. *Journal of The American Society of Nephrology*.

[31] Hu N., Westra J., Huitema M. G. (2009). Coexpression of CD177 and membrane proteinase 3 on neutrophils in antineutrophil cytoplasmic autoantibody-associated systemic vasculitis: anti-proteinase 3-mediated neutrophil activation is independent of the role of CD177-expressing neutrophils. *Arthritis and Rheumatism*.

[32] Jones R. B., Tervaert J. W. C., Hauser T. (2010). Rituximab versus cyclophosphamide in ANCA-associated renal vasculitis. *The New England Journal of Medicine*.

[33] Stone J. H., Merkel P. A., Spiera R. (2010). Rituximab versus cyclophosphamide for ANCA-associated vasculitis. *The New England Journal of Medicine*.

[34] Toubi E., Kessel A., Slobodin G. (2007). Changes in macrophage function after rituximab treatment in patients with rheumatoid arthritis. *Annals of the Rheumatic Diseases*.

[35] Díaz-Torné Cr, de Juana M. A. Ortiz, Geli C. (2014). Rituximab-induced interleukin-15 reduction associated with clinical improvement in rheumatoid arthritis. *Immunology*.

