# Supplementary material for: Disease Activity and Tendency to Relapse in ANCA-Associated Vasculitis Are Reflected in Neutrophil and Intermediate Monocyte Frequencies
Source: J Immunol Res. 2024 Jan 3;2024:6648265. doi: 10.1155/2024/6648265 (PMC10781522; doi:10.1155/2024/6648265)
Supplement: Supplementary Materials — Figure S1: gating strategy for granulocyte and monocyte analysis. Table S1: leukocytes frequency in MPA patients and healthy controls. Table S2: leukocytes frequency in GPA patients and healthy controls. Table S3: comparison of leukocytes in GPA and MPA patients. Table S4: comparison of leukocytes in MPO and PR3-positive AAV patients. Table S5: leukocytes frequency in patients in active disease and healthy controls. Table S6: leukocytes frequency in patients in remission and healthy controls. Table S7: comparison of leucocytes in AAV patients with and without RTX treatment. [file 6648265.f1.docx]

### Supplementary Materials

**Figure S1. Gating strategy for granulocyte and monocyte analysis**

Table S1 Leukocytes frequency in MPA patients and healthy controls

| **Cell type (% of WBC)** | **MPA patients (n=37)** | **HC (n=126)** | **p-value** |
| --- | --- | --- | --- |
| Basophils | 0.7 (0.03-2.3) | 0.7 (<0.001-2.5) | ns |
| Eosinophils | 2 (0.02-8) | 2.2 (0.2-16) | ns |
| Neutrophils CD16^dim^ | 1.9 (0.1-15.6) | 1.5 (0.008-10) | ns |
| Mature neutrophils | 42 (12-82) | 25 (3-63) | 0.0004 |
| Neutrophils CD177^+^ | 19 (0-70) | 11 (0-39) | 0.004 |
| Monocytes, total | 3.7 (0.8-22) | 3.8 (0.3-9) | ns |
| Classical monocytes | 3 (0.7-21) | 3.4 (0.3-9) | ns |
| Intermediate monocytes | 0.2 (0.04-1.2) | 0.1 (0-1.02) | 0.0002 |
| Non-classical monocytes | 0.2 (<0.001-0.8) | 0.2 (0.01-1) | ns |

Mann-Whitney *U* test was used to calculate level of significance. Data are presented with medians (ranges). For the basophil analysis 31 MPA patients and 67 HC were included. For eosinophil analysis 30 MPA patients and 83 HC were included. MPA: microscopic polyangiitis; HC: healthy controls; ns: not significant.

Table S2 Leukocytes frequency in GPA patients and healthy controls

| **Cell type (% of WBC)** | **GPA pat (n=68)** | **HC (n=126)** | **p-value** |
| --- | --- | --- | --- |
| Basophils | 0.6 (0.04–7.2) | 0.7 (<0.001-2.5) | ns |
| Eosinophils | 1.9 (<0.01-20) | 2.2 (0.2-16) | ns |
| Neutrophils CD16^dim^ | 2.3 (0.06-11) | 1.5 (0.008-10) | 0.003 |
| Mature neutrophils | 33.9 (4.8-81.6) | 25 (3.0-63) | 0.003 |
| Neutrophils CD177^+^ | 15.8 (2.4-67) | 11 (0-39) | 0.0002 |
| Monocytes, total | 4.4 (0.7-19) | 3.8 (0.3-9) | 0.005 |
| Classical monocytes | 3.9 (0.5-17.6) | 3.4 (0.3-8.9) | 0.008 |
| Intermediate monocytes | 0.2 (0.04–1.3) | 0.1 (0-1.02) | <0.0001 |
| Non-classical monocytes | 0.2 (0.01-0.9) | 0.2 (0.01-1) | ns |

Mann-Whitney *U* test was used to calculate level of significance. Data are presented with medians (ranges). For the basophil analysis 63 GPA patients and 67 HC were included. For eosinophil analysis 83 HC were included. GPA: granulomatosis with polyangiitis; HC: healthy controls; ns: not significant.

Table S3. Comparison of leucocytes in GPA and MPA patients

| **Cell type (% of WBC)** | **GPA (n=68)** | **MPA (n=37)** | **p-value** |
| --- | --- | --- | --- |
| Basophils | 1.0 (0.04-7.2) | 0.7 (0.03-2.3) | ns |
| Eosinophils | 1.4 (0-20) | 1.2 (0-8.1) | ns |
| Neutrophils CD16^dim^ | 2.3 (0.06-11) | 1.9 (0.1-16) | ns |
| Mature neutrophils | 34 (4.8-82) | 42 (12-82) | ns |
| Neutrophils CD177^+^ | 16 (2.4-67) | 19 (0-70) | ns |
| Monocytes, total | 4.4 (0.7-19) | 3.7 (0.8-23) | ns |
| Classical monocytes | 3.9 (0.5-18) | 3.1 (0.7-21) | 0.04 |
| Intermediate monocytes | 0.2 (0.04-1.3) | 0.2 (0.04-1.2) | ns |
| Non-classical monocytes | 0.2 (0.01-0.9) | 0.2 (0.003-0.8) | ns |

Mann-Whitney *U* test was used to calculate level of significance. Data are presented with medians (ranges). For basophil analysis 62 GPA patients and 30 MPA patients were included. For analysis of eosinophils 30 MPA patients were included. For analysis of classical monocytes 36 MPA patients were included. GPA: granulomatosis with polyangiitis; MPA: microscopic polyangiitis; WBC: white blood cell; ns: not significant.

Table S4. Comparison of leucocytes in MPO and PR3 positive AAV patients

| **Cell type (% of WBC)** | **MPO (n=40)** | **PR3 (n=60)** | **p-value** |
| --- | --- | --- | --- |
| Basophils | 0.8 (0.04-7.2) | 0.8 (0.04-2.6) | ns |
| Eosinophils | 1.5 (0-14) | 1.2 (0-20) | ns |
| Neutrophils CD16^dim^ | 1.8 (0.06-11) | 2.2 (0.1-16) | ns |
| Mature neutrophils | 38 (12-82) | 34 (9.5-82) | ns |
| Neutrophils CD177^+^ | 18 (0-70) | 17 (2.9-67) | ns |
| Monocytes, total | 4.7 (1.2-24) | 5.3 (1.2-15) | ns |
| Classical monocytes | 3.2 (0.5-21) | 4.1 (0.7-14) | ns |
| Intermediate monocytes | 0.2 (0.04-1.2) | 0.2 (0.04-1.3) | ns |
| Non-classical monocytes | 0.3 (0.003-0.9) | 0.2 (0.01-0.9) | ns |

Mann-Whitney *U* test was used to calculate level of significance. Data are presented with medians (ranges). For basophil analysis 34 MPO positive and 55 PR3 positive patients were included. For analysis of eosinophils 57 PR3 positive patients were included. MPO: myeloperoxidase; PR3: proteinase 3; AAV: anti-neutrophil cytoplasmic antibody (ANCA)-associated vasculitis; WBC: white blood cell; ns: not significant.

Table S5. Leukocytes frequency in patients in active disease and healthy controls

| **Cell type (% of WBC)** | **Active disease (n=14)** | **HC (n=126)** | **p-value** |
| --- | --- | --- | --- |
| Basophils | 0.4 (0.03-1.4) | 0.7 (<0.01-2.5) | ns |
| Eosinophils | 1.5 (0.7-20) | 2.2 (0.2-16) | ns |
| Neutrophils CD16^dim^ | 1.5 (0.06-8.9) | 1.5 (<0.01-10) | ns |
| Mature neutrophils | 34 (12-77) | 25 (3.0-63) | ns |
| Neutrophils CD177^+^ | 23 (<0.01-70) | 11 (<0.01-39) | 0.01 |
| Monocytes, total | 3.8 (0.7-11) | 3.8 (0.3-9) | ns |
| Classical monocytes | 3.4 (0.5-10) | 3.4 (0.3-8.9) | ns |
| Intermediate monocytes | 0.16 (0.04-0.5) | 0.1 (<0.01-1.02) | ns |
| Non-classical monocytes | 0.16 (0.003-0.9) | 0.2 (0.01-1) | ns |

Mann-Whitney *U* test was used to calculate level of significance. Data are presented with medians (ranges). For the basophil analysis 10 patients in active disease and 67 HC were included. For eosinophil analysis 8 patients in active disease and 83 HC were included. HC: healthy controls; ns: not significant.

Table S6. Leukocytes frequency in patients in remission and healthy controls

| **Cell type (% of WBC)** | **Remission (n=91)** | **HC (n=126)** | **p-value** |
| --- | --- | --- | --- |
| Basophils | 0.9 (0.04-7.2) | 0.7 (<0.01-2.5) | ns |
| Eosinophils | 2 (<0.01-14.3) | 2.2 (0.2-16) | ns |
| Neutrophils CD16^dim^ | 2.3 (0.14-16) | 1.5 (<0.01-10) | 0.001 |
| Mature neutrophils | 36 (4.7-82.3) | 25 (3.0-63) | 0.0001 |
| Neutrophils CD177^+^ | 16 (0-66) | 11 (0-39) | 0.0001 |
| Monocytes, total | 4.3 (0.9-23) | 3.8 (0.3-9) | 0.026 |
| Classical monocytes | 3.8 (0.7-21) | 3.4 (0.3-8.9) | ns |
| Intermediate monocytes | 0.2 (0.04-1.3) | 0.1 (<0.01-1.02) | <0.0001 |
| Non-classical monocytes | 0.2 (0.04-0.9) | 0.2 (0.01-1) | ns |

Mann-Whitney *U* test was used to calculate level of significance. Data are presented with medians (ranges). For the basophil analysis 84 patients in remission and 67 HC were included. For eosinophil analysis 84 patients in remission and 83 HC were included. HC: healthy controls; ns: not significant.

Table S7. Comparison of leucocytes in AAV patients with and without RTX treatment

| **Cell type (% of WBC)** | **RTX (n= 22)** | **No RTX (n= 83)** | **p-value** |
| --- | --- | --- | --- |
| Basophils | 0.8 (0.1-2.6) | 0,8 (0.03-7.2) | ns |
| Eosinophils | 2.4 (0.09-7.4) | 1.8 (0.007-20) | ns |
| Neutrophils CD16^dim^ | 1.8 (0.2-11) | 2.3 (0.06-16) | ns |
| Mature neutrophils | 40 (14-72) | 35 (4.8-82) | ns |
| Neutrophils CD177^+^ | 20 (2.7-43) | 16 (0-70) | ns |
| Monocytes, total | 5.5 (1.9-23) | 4 (0.7-16) | 0.02 |
| Classical monocytes | 4.7 (1.6-21) | 3.7 (0.5-15) | 0.03 |
| Intermediate monocytes | 0.3 (0.05-1.1) | 0.2 (0.04-1.3) | 0.03 |
| Non-classical monocytes | 0.3 (0.07-0.9) | 0.2 (0.003-0.9) | ns |

Mann-Whitney *U* test was used to calculate level of significance. Data are presented with medians (ranges). For basophil analysis 72 patients without RTX were included. For analysis of eosinophils 21 patients with RTX and 71 patients without RTX were included. AAV: anti-neutrophil cytoplasmic antibody (ANCA)-associated vasculitis; RTX: rituximab; WBC: white blood cell, ns: not significant.
